# Adult central nervous regeneration in *Drosophila*: Evidence for glial lineage conversion and neurogenic potential post-injury

**DOI:** 10.4103/NRR.NRR-D-25-00768

**Published:** 2025-11-25

**Authors:** Sergio Casas-Tintó, Maria Losada-Pérez

**Affiliations:** Instituto de Investigación de Enfermedades Raras, Instituto de Investigación Carlos III, Madrid, Spain; Departamento de Biología Celular e Histología, Universidad Complutense de Madrid, Madrid, Spain

Adult neurogenesis is generally considered to be very limited; however, there is increasing evidence that this phenomenon is conserved across species. Traditionally, research has focused on identifying precursor cells, those that are actively dividing or have the potential to divide. Direct evidence of adult neurogenesis has been found in rats, mice, songbirds, and nonhuman primates. In humans, while the evidence is indirect, it strongly suggests that neurogenesis also occurs during adulthood. In mammals, this active neurogenesis is preserved by radial glial progenitors, which remain in specific niches in the subventricular zone of the lateral ventricles and in the subgranular zone of the hippocampal dentate gyrus (Kumar et al., 2019).

It is currently believed that radial glial progenitors can revert to an immature, proliferative state, enabling them to divide and give rise to newborn neurons. In the study of adult neurogenesis, researchers commonly assess the presence of this process by analyzing cell proliferation and the expression of specific molecular markers associated with newly generated neurons (Kumar et al., 2019). However, recent results have shown that radial glial progenitors not only serve as precursor cells but also function as differentiated glia (Malatesta et al., 2008), suggesting that glial cells can shift from one differentiated fate to another, a process known as transdifferentiation. This challenges the long-standing notion of terminal differentiation in glial populations and opens exciting possibilities for regenerative biology and adult neurogenesis. In addition, similar glia-to-neuron transdifferentiation has also been reported in mammals. For instance, under specific injury or reprogramming conditions, astrocytes in the mouse cortex can be induced to adopt neuronal fates (Gong et al., 2022). Although these transformations were genetically induced, they suggest that glial plasticity is a conserved feature of the adult central nervous system (CNS).

To further understand the mechanisms and impact of these processes, there is a need for additional lineage tracing experiments. These studies would provide critical insights into the origin and fate of cells involved in CNS function and regeneration.

*Drosophila melanogaster* has emerged as a powerful model organism to investigate neurogenesis and glial plasticity, thanks to its extensive genetic toolkit and the high degree of conservation of key developmental and pathological pathways. In fact, findings in *Drosophila* often lay the groundwork for discoveries in vertebrate systems, with many concepts being validated years later in mammals (Bellen et al., 2010). An important example is the Notch signaling pathway, first genetically dissected in *Drosophila*, where its role in neural versus epidermal fate decisions was uncovered; this foundational work paved the way for understanding Notch’s conserved function in vertebrate neurogenesis, stem cell maintenance, and neurological disease (Bellen et al., 2010). Likewise, proneural genes such as Achaete-Scute complex-responsible for determining which cells in the developing nervous system become neurons- were initially described in *Drosophila*, and mammalian orthologs were described later as *Mash1* (*Ascl1*), showing equivalent functions in neural progenitors specification in the CNS during development and adult stages (Bellen et al., 2010)

**Drosophila in CNS regeneration research:** The adult CNS of *Drosophila melanogaster* has traditionally been viewed as non-neurogenic, with glial cells considered terminally differentiated and limited to supportive roles. However, recent studies have fundamentally challenged this notion. The development of novel injury paradigms in *Drosophila* has helped uncover key mechanisms of neural regeneration both during development and in the fully differentiated adult central nervous system (Fernandez-Hernandez et al., 2013; Harrison et al., 2021; Losada-Perez et al., 2021). CNS crush injury models in larvae have demonstrated dedifferentiation processes, showing changes in identities from glia to neuroblast (Harrison et al., 2021). Moreover, stab injury models in the adult brain have highlighted the existence of a mechanism that generates a transient stem cell activation zone (Simoes et al., 2022). Besides, controlled traumatic brain injury models in adult *Drosophila* have shown injury-induced changes in immune responses and metabolic adaptations (Saikumar et al., 2020; Li et al., 2024). Together, these diverse models demonstrate that the adult *Drosophila* CNS retains a significant degree of plasticity and that injury responses are shaped by both the nature of the damage and the regional context within the brain.

Following injury to the optic lobe, cellular damage responses such as Jun N-terminal kinase (JNK) and oxidative stress signaling activated by damage-responsive neuro-glial clusters might upregulate the expression of *Drosophila Myc* (*dMyc*) gene in adult neural progenitor cells, driving division and later generation of newborn neurons (Fernandez-Hernandez et al., 2013; Simoes et al., 2022).

**Figure 1:**
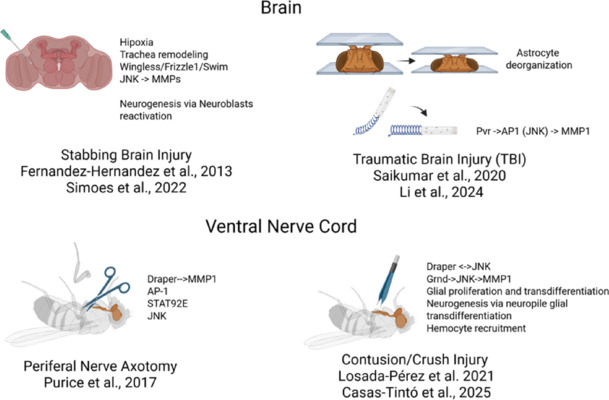
Schematic representation of different injury paradigms in the central nervous system of adult Drosophila. Both brain and ventral nerve cord injuries activate similar immune responses, whereas the neurogenic responses differ. Created with BioRender.com. AP-1: Activator protein 1; Grnd: Grindelwald; JNK: Jun N-terminal kinase; MMP: matrix metalloproteinase.

Our work using a crush injury model in adult *Drosophila* ventral nerve cord (VNC) has shown that neuropil glia possesses remarkable plasticity (Casas-Tinto et al., 2025). In normal conditions, ensheathing glia (EG), a subset of neuropil glia, can change their identity into astocyte-like glia, and both EG and astocyte-like glia are able to transdifferentiate directly into immature neurons, as evidenced by expression of the neuronal marker Elav. Moreover, this glia-to-neuron transformation is enhanced following injury. We also observed that astocyte-like glia cells divide in response to injury, whereas EG cells do not, revealing distinct cellular responses within the neuropil. This phenomenon represents a novel form of adult neurogenesis that occurs independently of traditional stem cell pathways. In contrast, a previous study by Fernández-Hernández et al. (2013), using a stab injury paradigm in the optic lobes, found quiescent neuroblasts – identified by Deadpan (Dpn) expression – that could be reactivated following acute brain injury, leading to proliferation and neurogenesis in the medulla cortex. These region-specific strategies underscore a broader concept: the adult *Drosophila* CNS possesses diverse regenerative capacities that vary by anatomical domain and cellular context (**Figure 1**).

Our earlier work (Losada-Perez et al., 2021) further supports the idea that glial cells coordinate a structured response to CNS damage, a phenomenon we and others have termed the glial regenerative response (GRR). In this model, EG and other glial types rapidly activate the JNK signaling pathway upon injury, engaging molecules such as the receptor Grindelwald (grnd) and the engulfment receptor Draper (drpr). Activation of these receptors facilitates debris clearance, modulates inflammatory responses, and promotes functional regeneration. We also demonstrated the recruitment of macrophage-like cells to the injury site, indicating a conserved and coordinated neuro-immune interaction that parallels aspects of vertebrate CNS repair.

It is tempting to postulate two different mechanisms for the regeneration of the brain and VNC. Following brain injury, dormant neural progenitors activate proliferative mechanisms and neuronal specification programs supported by neuro-glial clusters that sense the damage and trigger the response. In the VNC, there is no evidence of a neurogenic niche but there is of glial neurogenic response. In larvae, glial cells express the neuroblast marker *dpn* and lose their glial identity marker Repo, while in adult VNC, glial cells stop expressing the glial marker *repo* to directly express the neuronal marker *elav.* These two different mechanisms in different regions might reflect region-specific strategies for regeneration, tailored not only to the cellular composition and developmental history of each neural compartment, but also to their distinct functional requirements. While the brain relies on specialized neuro-glial clusters to reactivate dormant progenitors and support neurogenesis, the VNC appears to employ a direct glia-to-neuron conversion pathway, bypassing a canonical stem cell niche. This evidence highlights the remarkable plasticity of glial cells and underscores the diversity of regenerative programs within the central nervous system.

Together, these findings establish *Drosophila* as a powerful model for investigating the cellular and molecular mechanisms of adult CNS regeneration, while simultaneously challenging the traditional dichotomy between glial and neuronal lineages. Instead, they reveal a more fluid and dynamic relationship in which glial cells, under specific cues, can shift their roles and identities, potentially contributing to neural repair. The discovery of direct glia-to-neuron transdifferentiation in the adult VNC significantly expands our understanding of neural plasticity and underscores the remarkable capacity of glial cells to be reprogrammed *in vivo*. This opens new avenues for exploring how cellular identity is regulated and redefined within the adult nervous system. Future research will aim to elucidate the molecular mechanisms driving glial fate changes, identify the signals that initiate reprogramming, and determine how newly generated neurons functionally integrate into pre-existing neural circuits.

**Molecular parallelism between glial regenerative response and glioblastoma progression:** Our work in regeneration and in glial brain tumors has made it clear that GRR and glioblastoma (GB) progression share key molecular pathways that mediate glial proliferation, migration, and survival (Portela et al., 2019). However, while GRR is highly regulated and self-limiting, GB progression is a pathological condition generated by deregulated activation of the same mechanisms.

In *Drosophila melanogaster*, GRR to injury is extensively characterized at the molecular level. Upon mechanical injury, glial cells activate a gene network involving *Notch, kon-tiki, prospero, dorsal, relish, grindelwall, Kish*, and matrix metalloproteinases (*MMPs*) (homologous to *Notch1, NG2, Prox1, NFκB, TNFR, TMEM167A*, and *MMPs* in mammals). These mechanisms control proliferation and differentiation through feedback loops, balancing repair and preventing glioma (Losada-Perez et al., 2017, 2021).

This injury-induced proliferative response is transient, finishing once homeostasis is reestablished. By contrast, pathological glial proliferation models in *Drosophila* reveal how dysregulation of these same pathways can drive GB-like growth. Constitutive activation of epidermal growth factor receptor (EGFR) signaling (e.g., through expression of the oncogenic EGFR or RasV12 variant) and co-activation of the phosphoinositide 3-kinase (PI3K) pathway lead to aggressive overproliferation of glial cells, tumor microtube formation, infiltration, and loss of CNS architecture (Portela et al., 2019).

The combined activation of the PI3K and EGFR signaling pathways plays a critical role in neurogenesis and CNS regeneration. It has been shown that simultaneous stimulation of these pathways in glial cells transforms the otherwise inhibitory CNS environment, enabling unprecedented axon regrowth (Li et al., 2020). This transformation is associated with glial metabolic rewiring and architectural remodeling, which together support regenerative growth. Furthermore, cortex glial niche cells have been found to integrate both external nutritional cues and local neural stem cell activity, leading to dynamic remodeling of their architecture. This glial encapsulation of neural stem cell lineages is essential for effective neurogenesis, as it ensures the survival of newborn neurons. Selective manipulations of the PI3K/Akt pathway have revealed both cell-autonomous and non-cell-autonomous signals necessary for the formation of neurogenic niches. These glial niches begin to form around the time of the first neural stem cell division, prior to substantial neuronal production, highlighting their critical role in supporting early neurogenic processes (Li et al., 2020).

In contrast, constitutive activation of the EGFR and PI3K pathways in glial cells serves as a key oncogenic trigger for GB formation. In these tumor models, glial cells undergo uncontrolled proliferation and generate highly invasive, actin-based membrane extensions known as tumor microtubes. These tumor microtubes are not only structural components that enable the tumor to infiltrate healthy brain tissue, but they also establish tight, functional interactions between GB cells and neurons. This intimate GB-to-neuron communication facilitates the hijacking of neuronal signals, particularly through the deregulation of WNT/Wingless signaling. As described by Portela et al. (2019), this imbalance leads to the persistent activation of the JNK pathway and an upregulation of MMPs, which occurs independently of external injury. The sustained JNK signaling reinforces the invasive phenotype of GB cells and promotes tissue degradation via MMPs, further enhancing tumor progression and resistance to conventional therapies. This cascade underscores the pathological reprogramming of glial cells into malignant entities through aberrant EGFR and PI3K signaling, illustrating the stark contrast between physiological neurogenic remodeling and oncogenic transformation.

Thus, results in *Drosophila* models suggest a balance between physiological glial proliferation in response to injury and pathological tumorigenic proliferation. In the regenerative context, glial cells activate mitogenic pathways transiently and are subject to feedback inhibition and differentiation cues that terminate the repair process. In GB models, these feedback mechanisms are disabled, and mitogenic signaling remains constitutively active, leading to neoplastic growth. This difference in regulatory mechanisms that mediate GB represents a pathological condition, proposed here as a disruption of regenerative programs.

**Concluding remarks:** Recent findings in *Drosophila* melanogaster challenge the long-standing view of the adult CNS as a non-regenerative system. Evidence from both the brain and ventral nerve cord reveals unexpected plasticity of glial cells, including their ability to transdifferentiate into neurons in response to injury. This phenomenon highlights alternative, region-specific regenerative strategies: while neural progenitors can be reactivated in the brain, glial cells in the VNC engage in direct fate conversion, bypassing classical stem cell pathways. These discoveries reshape our understanding of glial identity and function in the adult CNS and underscore the value of Drosophila as a model for dissecting the cellular and molecular logic of regeneration. Furthermore, the parallels between glial regenerative responses and glioblastoma progression emphasize the need to understand how tightly regulated repair mechanisms can become pathological when deregulated. Together, these insights open promising avenues for future research in regenerative biology and neural repair.

*This work was supported by MCIN/ AEI (Spanish Government), Grant No. PID2022-137751OA-00I (to MLP).*
